# The Ghrelin-LEAP2 System in Obesity and Diabetes: Pathophysiological Roles and Therapeutic Potential

**DOI:** 10.1007/s13679-026-00728-1

**Published:** 2026-06-12

**Authors:** Isabela Valdés-Calero, Gema Frühbeck, Amaia Rodríguez

**Affiliations:** 1 Department of Endocrinology and Nutrition, Clínica Universidad de Navarra, Avda. Pío XII 36, 31008 Pamplona, Spain; 2 Metabolic Research Laboratory, Clínica Universidad de Navarra, Irunlarrea 1, 31008 Pamplona, Spain; 3 CIBER de Fisiopatología de La Obesidad y La Nutrición (CIBEROBN), Instituto de Salud Carlos III, Madrid, Spain; 4 Obesity and Adipobiology Group, Instituto de Investigación Sanitaria de Navarra (IdiSNA), Pamplona, Spain; 5 Institute for Nutrition and Health, University of Navarra, Pamplona, Spain

**Keywords:** Ghrelin, LEAP2, GHSR, Obesity, Type 2 diabetes

## Abstract

**Purposeof Review:**

Ghrelin is a gut-derived acylated hormone that regulates appetite, food reward-related behaviours, glycaemic control, and lipid metabolism. These actions are primarily mediated through activation of the growth hormone secretagogue receptor (GHSR), which is highly expressed in the brain and also present in key metabolic organs. Ghrelin acylation by ghrelin *O*-acyltransferase (GOAT) is required for receptor binding and biological activity. This review examines the physiological and pathophysiological roles of ghrelin and liver-expressed antimicrobial peptide 2 (LEAP2), with particular emphasis on its relevance to obesity and type 2 diabetes.

**Recent Findings:**

LEAP2 has recently been identified as an endogenous antagonist and inverse agonist of GHSR that counteracts ghrelin signalling. Emerging evidence indicates that obesity and type 2 diabetes are generally associated with reduced circulating ghrelin levels and increased LEAP2 concentrations, particularly in the presence of insulin resistance. These reciprocal changes support the concept that the ghrelin/LEAP2 ratio functions as a dynamic regulator of energy balance, glucose homeostasis, and appetite control, and may influence metabolic responses to nutritional status and weight loss.

**Summary:**

The ghrelin-LEAP2 system represents a key regulatory pathway in metabolic homeostasis and a promising therapeutic target for obesity and related metabolic disorders. Pharmacological strategies targeting this axis, including ghrelin antagonists, LEAP2 analogues, GOAT inhibitors, and GHSR inverse agonists, are under active investigation, although further studies are required to establish their long-term efficacy and safety.

## Introduction

Discovered in 1999, ghrelin (from ghre, the Proto-Indo-European root meaning “grow”) is an acylated peptide predominantly secreted by enteroendocrine cells in the stomach and duodenum [1, 2]. Ghrelin was identified as the endogenous ligand of the growth hormone (GH) secretagogue receptor (GHSR), mediating GH release from the anterior pituitary [1]. GHSR is a member of the seven transmembrane G protein-coupled receptor family, and is expressed in multiple brain regions, including the hypothalamus and pituitary [3], as well as in several pancreatic cell types (α, β, δ and acinar cells) [4, 5], throughout the gastrointestinal tract (stomach, intestine and vagal afferent neurons) [6], and in the adipose tissue [7]. This broad expression underscores the central role of ghrelin-GHSR signalling in energy homeostasis. To date, ghrelin has been implicated in multiple metabolic processes, including meal initiation [8], increased food intake and adiposity [9], reduced energy expenditure [10, 11], and maintenance of blood glucose levels during starvation to prevent hypoglycaemia [12–14]. It also modulates reward-related behaviours toward food, alcohol, and addictive drugs [15, 16], among others. Notably, circulating ghrelin concentrations are generally reduced in metabolic disorders such as obesity [17], type 2 diabetes (T2D) [18], polyendocrine metabolic ovarian syndrome (PMOS) [19] and metabolic dysfunction-associated steatotic liver disease (MASLD) [20, 21].

Liver-expressed antimicrobial peptide 2 (LEAP2) was first isolated in 2003 from human blood and characterized as a cationic antimicrobial peptide with protective functions against bacterial infection [22]. LEAP2 is primarily expressed in the liver and jejunum. Hepatic expression is moderate and restricted to hepatocytes, whereas in the jejunum it is highly enriched in villus enterocytes, with little to no expression in the lamina propria or crypts [23, 24]. Originally, LEAP2 was classified as an antimicrobial peptide because its plasma concentrations rise during illness [25, 26]. However, its physiological relevance expanded markedly in 2018, when LEAP2 was discovered to act as an endogenous antagonist of GHSR [25, 26]. Plasma LEAP2 concentrations decline during energy-deficient states [23] and augment in obesity [27], serving as a physiologically relevant signal that regulates GHSR activity. Specifically, LEAP2 acts both as a GHSR antagonist and an inverse agonist, thereby blocking the effects of ghrelin and suppressing the constitutive activity of the receptor [23, 28]. Administration of LEAP2 reduces spontaneous, ghrelin-stimulated, and high-fat-diet (HFD)-induced food intake, lowers blood glucose levels, and suppresses both ghrelin-induced and postprandial GH secretion [23, 29, 30]. In metabolic disorders, LEAP2 concentrations change in opposite direction to ghrelin, being increased in obesity [27], T2D [31], or MASLD [32].

This Review synthesizes current evidence on the physiology, regulation, and systemic metabolic actions of the ghrelin-LEAP2 system, and extends this framework to pathophysiological conditions such as obesity and type 2 diabetes. Integration of findings from basic, translational, and clinical studies highlights how the dynamic interplay between ghrelin and LEAP2 contributes to metabolic homeostasis and disease. The therapeutic potential and safety of targeting this hormonal axis are also considered, underscoring key priorities for future research.

## Regulation and Physiology of the Ghrelin-LEAP2 System

### Biosynthesis and Post-Translational Processing of Ghrelin and LEAP2

**Fig. 1:**
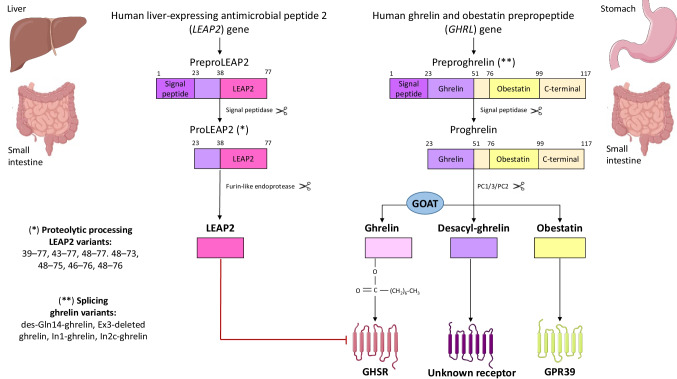
Human ghrelin and LEAP2 processing. Liver-expressed antimicrobial peptide 2 (LEAP2) is a peptide secreted by hepatocytes and enterocytes. Human *LEAP2* gene encodes the 77-amino-acid preproLEAP-2, which is first cleaved by signal peptidase to generate proLEAP-2, which is subsequently processed by a furin-like endoprotease to produce the 40-residue mature LEAP-2 (corresponding to residues 38–77). Mature LEAP-2 may undergo further proteolytic cleavage into smaller metabolites. This mature isoform is the predominant circulating variant and acts as an antagonist of the ghrelin receptor (GHSR). In contrast, the human ghrelin gene (*GHRL*) encodes a 117-amino-acid precursor, preproghrelin, which gives rise to several biologically active peptides produced by endocrine cells of the stomach and small intestine. Preproghrelin is cleaved by signal peptidase to generate the 94-amino-acid proghrelin, most of which remains unacylated (desacyl ghrelin). A smaller fraction undergoes post-translational acylation at serine-3 by ghrelin O-acyltransferase (GOAT), yielding the active, *n*-octanoylated ghrelin. Ghrelin and obestatin exert their biological effects through GHSR and GPR39, respectively, whereas the receptor for desacyl ghrelin has not yet been identified. Several alternative GHRL transcripts have been detected in various cancer types, highlighting the complex transcriptional regulation of this gene

The human ghrelin gene (*GHRL*), located at chromosome 3p26-p25, encodes a 117-amino-acid polypeptide known as preproghrelin, comprising a 23–amino-acid signal peptide and a 94–amino-acid proghrelin segment (Fig. 1). After translocation to the endoplasmic reticulum (ER), preproghrelin undergoes processing to generate proghrelin and obestatin [33, 34]. Within the ER, the acyltransferase ghrelin *O*-acyltransferase (GOAT) catalyzes the addition of an octanoic acid moiety to serine-3 of proghrelin, a modification required for producing the biologically active ghrelin [35, 36]. Following acylation, acyl-proghrelin is transported to the Golgi apparatus, where prohormone convertase 1/3 (PC1/3) or PC2 cleave proghrelin to release mature ghrelin [34, 37]. Both ghrelin and desacyl-ghrelin isoforms are subsequently packaged into secretory vesicles and released into the circulation [38]. To date, it remains unclear whether desacyl-ghrelin arises from incomplete acylation of the proghrelin peptide or from the deacylation of mature ghrelin [39]. In plasma, desacyl-ghrelin represents approximately 90% of total circulating ghrelin, while the acylated, active form accounts for roughly 10% [7, 38, 40]. Aberrant splicing of *GHRL* gene can lead to the production of oncogenic variants, such as the intron 1-retention ghrelin (In1-ghrelin variant), which is overexpressed in specific cancer types, including breast, prostate, pituitary, and neuroendocrine tumours [41–43]. In accordance with the recent nomenclature consensus [44], the term “ghrelin” is used throughout this review to refer to acylated ghrelin unless otherwise specified, whereas the non-acylated form is referred to as “desacyl-ghrelin”.

The human LEAP2 gene (*LEAP2*), located at chromosome 5q31, encodes a 77-amino-acid preproprotein composed of a 22-amino-acid signal sequence and a 53 amino-acid propeptide (Fig. 1) [24]. During maturation, the propeptide is cleaved by the proprotein convertase furin to generate the mature form of LEAP2, a 40-amino-acid bicyclic peptide (corresponding to residues 38–77) containing two disulfide bridges and exhibiting both antimicrobial and metabolic activities [22, 45]. Several additional circulating mature forms with variable N-terminal length have also been identified, including the 39–77, 43–77, 48–77, 48–73, 48–75, 46–76, and 48–76 variants, highlighting a degree of structural heterogeneity. The longest isoform, LEAP2_38–77_, contains a characteristic basic amino acid cluster, –RKRR– (positions 66–69), which is typical of many peptides with antimicrobial activity [22]. Because LEAP2-producing cells lack a regulated secretory pathway, its plasma levels are primarily determined by the rate of peptide biosynthesis [46].

### Dynamic Interplay of Circulating Ghrelin and LEAP2

As endogenous ligands of GHSR, ghrelin and LEAP2 act in concert to fine-tune GHSR activity in response to circadian and metabolic cues [47]. Circulating ghrelin levels display a clear circadian rhythmicity; however, this rhythm is influenced by meal timing, sleep patterns and stress. Ghrelin is tightly regulated by feeding status, rising approximately twofold before a meal and falling back to pre-fasting levels 1 h after eating [8]. The macronutrient composition of the meal influences the extent of this postprandial suppression, which is more pronounced following carbohydrate- and protein-rich meals than after fat-rich meals [48]. The circadian pattern of ghrelin is also linked to sleep–wake cycles; when sleep is misaligned or fragmented (e.g. night-time snacking, night eating syndrome, chronic night shift workers or persistent jetlag), ghrelin levels increase and correlate with increased appetite [47, 49, 50]. Consistent with this, ghrelin expression and secretion are directly governed by the suprachiasmatic nucleus (SCN), the master circadian clock, and peripheral clock mechanisms [49, 51]. The SCN, a small nucleus within the hypothalamus located just above the optic chiasm, receives indirect photic input from the retina and orchestrates circadian rhythms that synchronize systemic physiology. Single-cell RNA-sequencing studies have identified several SCN neuronal clusters with distinct spatial distributions, circadian rhythmicity, and responsiveness to light-mediated retinal input [52]. Furthermore, a population of GHSR-expressing neurons with indirect light responsiveness has recently been described in the murine SCN; these neurons regulate eating behaviour, feed efficiency, and body weight in a manner dependent on the mid-rest phase of the circadian cycle [51]. In addition, melatonin, a pineal hormone involved in circadian rhythm regulation, has been reported to modulate ghrelin secretion both directly and indirectly through circadian regulatory pathways. Experimental studies in preclinical models indicate that melatonin supplementation can suppress circulating ghrelin levels and downregulate *Ghrl* gene expression, effects associated with reduced food intake [53–55]. These observations further support the close interaction between circadian regulation and ghrelin-mediated metabolic control. Stress has also a relevant impact on ghrelin concentrations, with both acute and chronic stress elevating ghrelin levels, which may contribute to stress-related eating behaviours [56–58].

In healthy people, circulating LEAP2 concentrations are substantially higher (in the nanomolar range) than those of ghrelin (in the picomolar to low-nanomolar range) [25, 27, 31, 59]. Even though LEAP2 is not as extensively characterized as ghrelin, its circulating levels display an inverse regulatory pattern, declining during periods of energy deprivation and rising after refeeding [23, 27, 29]. LEAP2 concentrations also seem sensitive to intestinal exposure to specific substrates, such as glucose, lactate, β-hydroxybutyrate, and oleic acid, underscoring the need for further studies on the link between nutrient sensing and LEAP2 regulation [60, 61]. Postprandial increases in plasma LEAP2 fluctuate more gradually (fluctuations of ∼15% to 25% 2-h after food intake) compared to other peptide hormones [46, 62]. Moreover, postprandial LEAP2 levels correlate positively with metabolic markers, suggesting that blood glucose, insulin, and/or triglycerides may contribute to stimulating LEAP2 secretion [27, 62–65]. In line with this, it has been proposed that the liver, rather than the duodenum or jejunum, may be the principal source of postprandial LEAP2 release [66]. This opposing modulation of ghrelin and LEAP2 in response to nutritional status is consistent with the role of LEAP2 as an endogenous GHSR antagonist that counterbalances ghrelin signalling.

## Mechanistic Basis of Energy Balance Regulation by the Ghrelin-LEAP2 System

### Central Regulation of Food Intake

**Fig. 2:**
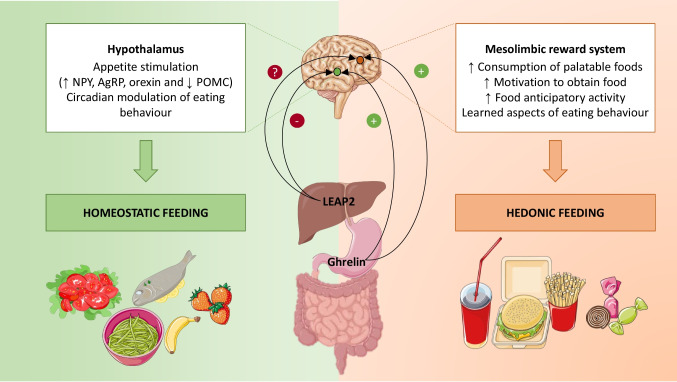
Central actions of ghrelin and LEAP2 on homeostatic and hedonic feeding. Ghrelin is secreted preprandially from the stomach and regulates homeostatic feeding by activating hypothalamic neurons expressing the orexigenic peptides NPY, AgRP, and orexin, while concurrently inhibiting neighbouring anorexigenic POMC neurons. In addition, GHSR-positive neurons recently identified in the suprachiasmatic nucleus—the master circadian clock located in the hypothalamus—exhibit light-responsive activity, providing mechanistic insight into the circadian regulation of feeding behaviour. Beyond homeostatic regulation, ghrelin also drives hedonic feeding by engaging mesolimbic reward pathways that support goal-directed behaviour toward palatable foods, food anticipation, and food-motivated actions. By contrast, LEAP2 is predominantly produced by the liver in the postprandial phase and reduces food intake by counteracting ghrelin. LEAP2 promotes appetite suppression partly by increasing the activity of hypothalamic POMC neurons. LEAP2 is also expressed in reward-related brain regions, although its contribution to the regulation of palatable food intake remains insufficiently understood and requires further investigation

Food intake is controlled by two interconnected drives: the homeostatic and hedonic pathways [67]. Under most physiological conditions, eating is not initiated solely by acute energy depletion, but is strongly influenced by learned associations, environmental cues, emotional states, and sensory stimuli. The homeostatic system preserves energy balance by enhancing the drive to eat when energy stores are depleted, whereas hedonic feeding is motivated by the rewarding and pleasurable properties of food. Dysregulation of these neurocircuits contributes to pathological states of hypophagia (e.g. anorexia nervosa or cachexia) and hyperphagia (e.g. obesity, Prader-Willi syndrome or eating disorders). Understanding the mechanisms underlying the interaction between homeostatic and hedonic circuits is essential for identifying new therapeutic targets for metabolic diseases. In this context, ghrelin engages multiple brain circuits to modulate both homeostatic and hedonic feeding (Fig. 2) [68]

#### Hypothalamus (Homeostatic Feeding)

The hypothalamus serves as a hub integrating diverse peripheral signals, including adipokines, gut hormones, and circulating nutrients, to precisely control food intake and maintain energy homeostasis. Several hypothalamic nuclei, including the arcuate (ARC), ventromedial (VMH), dorsomedial (DMH), paraventricular (PVH), the lateral hypothalamic area (LHA) regions and the master circadian clock SCN, express GHSR and play major roles in mediating ghrelin-induced effects on energy metabolism [51, 69, 70].

During fasting and the preprandial state, ghrelin is secreted from gastric enteroendocrine cells into the circulation, serving as a hormonal signal for hunger [71]. In humans, exogenous ghrelin administration at doses designed to mimic physiological or modestly supraphysiological preprandial increases has been shown to enhance subjective hunger and increase food intake, supporting its orexigenic role [72, 73]. However, whether endogenous physiological fluctuations in ghrelin alone are sufficient to trigger feeding remains debated, since *Ghrl-* and *Ghsr-*knockout models do not show hypophagic behaviour or major alterations in food intake compared with wild-type controls [74–76]. Ghrelin acts on energy-sensing Agouti-related peptide (AgRP)/neuropeptide Y (NPY)-expressing neurons in the ARC, which are particularly enriched in GHSR [77, 78]. Activation of these neurons by ghrelin stimulates the release of orexigenic peptides, including NPY, AgRP, and orexin, promoting food intake while concomitantly reducing energy expenditure [9, 79, 80]. Simultaneously, ghrelin uses inhibitory neurotransmitters such as γ-aminobutyric acid (GABA) to suppress neighbouring anorexigenic proopiomelanocortin (POMC) neurons [81]. At the intracellular level, ghrelin stimulates intracellular Ca^2^⁺ mobilization and activates the sirtuin-1 (SIRT1)/p53 pathway, AMP-activated protein kinase (AMPK), and the mammalian target of rapamycin (mTOR), ultimately promoting appetite stimulation in ARC neurons [82–85]. In the VMH, the orexigenic effects of ghrelin occur through activation of hypothalamic AMPK and inhibition of acetyl-CoA carboxylase (ACC) and fatty acid synthase (FASN), leading to decreased malonyl-CoA levels, increased carnitine palmitoyl-transferase 1 (CPT1) activity, and enhanced mitochondrial reactive oxygen species (ROS) production [86]. GHSR-expressing glucose-responsive neurons in the PVH, LHA, and DMH also contribute to the regulation of appetite, food reward, and neuroendocrine function in response to ghrelin [87, 88]. As noted earlier, GHSR-positive neurons recently identified in the SCN display light-responsive activity, providing mechanistic insight into the circadian modulation of feeding behaviour [51].

By contrast, LEAP2 reduces NPY/AgRP neuronal activity [27] and increases POMC neuronal activity [89], promoting appetite suppression and reducing body weight under both chow diet feeding and obesogenic conditions. Although chronic intracerebroventricular infusion of native LEAP2 decreases body weight and ghrelin-induced weight gain [30, 90], these findings should primarily be considered proof-of-concept pharmacological effects, as the physiological relevance of endogenous central LEAP2 signalling remains uncertain. Collectively, ghrelin and LEAP2 function as a dynamic, bidirectional regulatory system in which their relative concentrations modulate hypothalamic GHSR activity according to the nutritional state.

#### Mesolimbic Reward System (Hedonic Feeding)

Beyond its homeostatic actions in the hypothalamus, ghrelin also modulates mesolimbic dopamine pathways that govern food reward, motivation, and reinforcement. Ghrelin contributes to reward-based eating behaviour, with studies suggesting the role of ghrelin signalling in stress-induced alterations in feeding [91]. Ghrelin has consistently been shown to promote the consumption of palatable, energy-dense foods [91], increasing motivated behaviour for sugar [92–94] and fat [95]. In this sense, GHSR is expressed in multiple brain regions implicated in reward and motivation, including the ventral tegmental area (VTA), *nucleus accumbens*, amygdala, dorsal raphe nucleus (DRN) and hippocampus [96–98]. LEAP2 is broadly expressed in the brain, particularly in reward-related regions such as the laterodorsal tegmental area [99]. However, findings on the impact of LEAP2 on food preference remain mixed, with some studies reporting inhibitory actions on the palatable food intake and others showing no significant changes [99, 100].

The VTA represents a key node for motivated behaviours (including feeding), and have a population of GHSR-expressing neurons. Peripheral ghrelin can cross the blood–brain barrier through saturable bidirectional transport systems and may thereby access the VTA [101]. However, because the VTA is located deeper within the brain parenchyma than circumventricular organs lacking a tight blood–brain barrier, whether circulating ghrelin reaches VTA neurons at physiologically relevant concentrations remains uncertain [102]. Consequently, a substantial proportion of ghrelin-mediated effects on reward-related behaviour may occur indirectly through interconnected hypothalamic and brainstem pathways. On binding VTA neurons, ghrelin promotes motivation, increases appetite for palatable foods, and amplifies reward-seeking behaviors [97]. Ghrelin binding to VTA dopaminergic neurons increases their activity, promotes synaptic formation, and enhances dopamine turnover in the *nucleus accumbens* in rodents [97, 103]. These effect can be dampened by LEAP2 through GHSR antagonism in the VTA and *nucleus accumbens* [90]. In humans, ghrelin administration in healthy volunteers during functional magnetic resonance imaging enhances neural responses to food cues in multiple brain regions involved in hedonic feeding, including the amygdala, orbitofrontal cortex, hippocampus, striatum, and VTA [96]. In rodents, ghrelin administration into the DRN and hippocampus increases food intake, enhances memory retention, and modulates anxiety-like behaviour [98]. The basolateral amygdala represents a critical limbic region involved in the acquisition and storage of emotional memories. GHSR signalling within amygdala circuits shapes emotional memory processes, with ghrelin infusion into the amygdala improving memory retention and blocking conditioned taste aversion in rat [98, 104]. Conversely, LEAP2 infusion into the lateral amygdala abolishes the inhibitory effect of increased GHSR activity on conditioned taste aversion memory extinction [105].

Taken together, while strong evidence supports a key role for ghrelin in controlling hedonic feeding, current data on LEAP2 remain scarce and require further investigation.

### Peripheral Actions in Energy and Metabolic Control

**Fig. 3:**
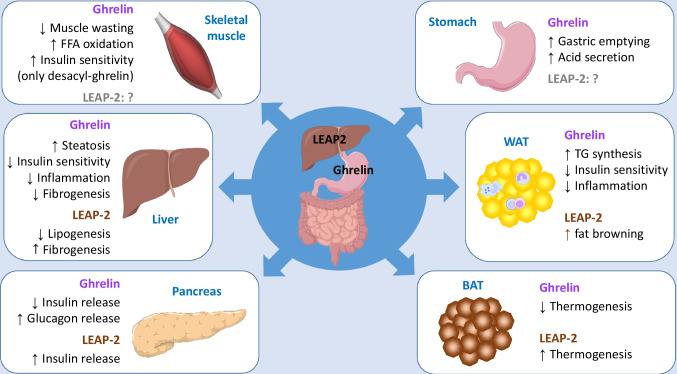
Peripheral actions of ghrelin and LEAP2 regulating energy and glucose homeostasis. Ghrelin promotes energy storage by increasing lipid accumulation in the white adipose tissue (WAT) and the liver, while reducing thermogenesis in both WAT and brown adipose tissue (BAT). Despite these lipogenic actions, ghrelin can also stimulate fatty acid oxidation in skeletal muscle and liver, and attenuate inflammation in adipose tissue and liver, thereby protecting these organs against lipotoxicity. In addition, ghrelin raises blood glucose through multiple mechanisms: (i) direct actions on pancreatic α- and β-cells, stimulating glucagon secretion and inhibiting glucose-induced insulin release, respectively; (ii) enhancement of hepatic gluconeogenesis; and (iii) reduced glucose uptake and insulin sensitivity in skeletal muscle and adipose tissue. In contrast, LEAP2 promotes energy expenditure by increasing thermogenesis in WAT and BAT. Its hepatic role remains incompletely understood, as it reduces lipogenesis in hepatocytes but also exerts mild profibrogenic effects on hepatic stellate cells

In addition to their central effects regulating food intake and energy homeostasis, ghrelin and LEAP2 also exert complementary and often opposing peripheral actions. Ghrelin promotes energy storage by stimulating lipid accumulation in adipose tissue, liver, and pancreas [7, 106–110], while suppressing thermogenesis in both WAT and BAT [111]. By contrast, LEAP2 acts as a counter-regulatory factor, promoting energy expenditure through increased thermogenesis in both fat depots [90]. Its hepatic effects are more complex, with both anti-lipogenic and mild profibrogenic actions [31, 112, 113]. Some of these effects are mediated directly through GHSR expressed in peripheral tissues, whereas others occur indirectly through activation of sympathetic and/or parasympathetic neural pathways. The integrated actions of ghrelin and LEAP2 on multiple organs are exposed in this section and summarized in Fig. 3.

#### Gastrointestinal Tract

Ghrelin also plays an important role in the regulation of gastrointestinal motility and gastric acid secretion, thereby preparing the digestive system for food intake. These effects are mediated primarily via activation of the vagal nerve, as pretreatment with atropine or bilateral cervical vagotomy abolishes ghrelin-induced gastric motility and acid secretion in rats [114, 115]. In humans, intravenous administration of ghrelin stimulates gastric emptying in healthy volunteers; this effect is independent of GH or motilin signaling and is likely mediated through vagal pathways [116]. Owing to its prokinetic properties, ghrelin agonists have been proposed as potential therapeutic agents for conditions characterized by impaired gastric motility, such as gastroparesis [117] and post-surgery gut slowdown [118]. To date, whether LEAP2 counteracts these gastrointestinal actions of ghrelin remains unknown.

#### Adipose Tissue

The human white adipose tissue (WAT) expresses ghrelin, GOAT, and the receptors for ghrelin-related peptides GHSR and GPR39 [7, 119, 120]. Ghrelin directly influences WAT metabolism by promoting lipid uptake, enhancing fat storage, and exerting anti-lipolytic effects. During adipogenesis, *GHRL* gene expression increases, and ghrelin-related peptides (ghrelin, desacyl-ghrelin and obestatin) upregulate key transcription factors involved in this process, including peroxisome proliferator-activated receptor γ (PPARγ) and sterol regulatory element-binding transcription factor 1 (SREBF1) [7, 106, 121]. Ghrelin, desacyl-ghrelin and obestatin also increase intracellular triacylglycerol accumulation by enhancing the expression of lipid uptake and storage-related proteins (lipoprotein lipase (LPL), ACC, FASN, and perilipin) through both central mechanisms [9, 86, 109, 122, 123] and direct actions on adipocytes [7, 124]. Ghrelin additionally facilitates lipid uptake into WAT via endothelial GHSR activation [125]. Furthermore, ghrelin-related peptides contribute to adipocyte hypertrophy by attenuating isoproterenol-induced lipolysis [126–131] and by enhancing insulin sensitivity [129, 132]. In contrast, leptin, an adipokine with opposing anorexigenic and lipolytic effects [133–136], suppresses *Ghrl* mRNA expression in human visceral adipocytes [120], highlighting reciprocal regulatory crosstalk between these hormonal systems.

Ghrelin also negatively regulates brown adipose tissue (BAT) thermogenesis by suppressing the sympathetic nervous system activity and repressing genes involved in thermogenesis, such as uncoupling protein 1 (UCP1), thereby shifting whole-body metabolism toward energy storage rather than heat production [111, 137]. Consistent with this, genetic suppression of GHSR activates BAT function and decreases fat storage in rats [138], supporting the notion that blocking GHSR signalling may represent a therapeutic strategy to combat obesity by enhancing BAT thermogenesis. In line with this concept, LEAP2 reduces adiposity by activating thermogenic programs in both WAT and BAT [90]. In addition, leptin supplementation combined with long-term antagonism of the ghrelin receptor using palm-LEAP2(1–14) exerts a beneficial and synergistic effect on rectal temperature and BAT uptake of glucose, the primary fuel source for thermogenesis, in leptin-deficient obese *ob/ob* mice [139].

#### Liver

The ghrelin system promotes a complex and context-dependent regulation of hepatic lipid metabolism by modulating signalling pathways involved in both lipid accumulation and lipid clearance [140]. Ghrelin and desacyl-ghrelin stimulate hepatic steatosis through the direct activation of hepatocytes upregulating several transcription factors related to hepatic lipogenesis, such as PPARγ, SREBF1 or CCAAT/enhancer-binding protein α (C/EBPα), as well as by upregulating key enzymes involved in triacylglycerol synthesis, such as monoacylglycerol O-acyltransferase 2 (MOGAT2) and diacylglycerol O-acyltransferase 1 (DGAT1) [107–109]. However, in parallel, ghrelin and obestatin activate AMPK in hepatocytes, triggering mitochondrial free fatty acid (FFA) β-oxidation [107, 141, 142]. Moreover, ghrelin activates lipophagy and restores circadian clock disruption in steatotic liver, actions that collectively limit lipotoxicity and preserve hepatocellular function [20, 107, 141–144]. In addition, ghrelin and desacyl-ghrelin act as potent anti-inflammatory agents in the liver by reducing pro-inflammatory cytokines (like TNF-α), inhibiting NF-κB signalling and programmed hepatocyte cell death, and suppressing hepatic stellate cell activation [20, 31, 145, 146]. Given its anti-apoptotic and anti-oxidative properties during liver injury, ghrelin has been suggested as a potential therapeutic target for metabolic dysfunction-associated steatohepatitis and other chronic liver diseases [140].

Ghrelin administration in rodents suppresses hepatic LEAP2 expression by an AMPK-dependent pathway [29]. However, the specific role of LEAP2 in hepatic lipid metabolism remains unclear, as *Leap2* knockdown has been associated with either an amelioration [63, 112] or an exacerbation [63, 147] of hepatic steatosis. A recent preclinical study demonstrated that LEAP2 decreases the expression of key de novo lipogenesis regulators in steatotic hepatocytes, including FASN, ACC, SREBF1, and carbohydrate-responsive element-binding protein (ChREBP), although this effect is blunted in obesity and ageing [112]. It has been also reported that hepatic LEAP2 expression is increased in MASLD through mechanisms that appear independent of classical insulin-, leptin-, or glucose-driven pathways [148, 149], suggesting a more complex regulatory control. In this regard, LEAP2 exerts a mild profibrogenic action by directly targeting hepatic stellate cells, and its hepatic expression increases during MASLD-associated fibrosis progression [31] as well as in alcohol-related liver disease [148].

#### Skeletal Muscle

Ghrelin plays an important role in skeletal muscle metabolism by promoting FFA oxidation, protecting against muscle atrophy, and supporting muscle regeneration. Both ghrelin and desacyl-ghrelin directly enhance muscle metabolic function and counteract muscle wasting in experimental models [150, 151], while alterations in circulating ghrelin profiles have been proposed as early indicators of sarcopenic obesity in elderly adults [152]. Consistent with these actions, anamorelin, a GHSR agonist, improves appetite and increases muscle specific mass in patients with cancer cachexia [153–155]. Mechanistically, ghrelin and desacyl-ghrelin stimulate mitochondrial FFA β-oxidation in skeletal muscle from rodents [156–158]. By enhancing lipid oxidation, ghrelin forms may limit the accumulation of lipotoxic intramuscular lipid intermediates and, thereby, reduce insulin resistance, a key contributor to T2D. However, skeletal muscle appears to become less responsive to desacyl-ghrelin under chronic HFD feeding, suggesting a state of “desacyl-ghrelin resistance” analogous to resistance to other metabolic hormones such as insulin, leptin, or adiponectin [157]. To date, the effects of LEAP2 on skeletal muscle metabolism and function remain unexplored.

### Ghrelin-LEAP2 Ratio: Nutritional State, Obesity, and Eating Disorders

The regulation of appetite and feeding behavior is modulated by several mechanisms. These include the ghrelin-LEAP2 system and the activity of the GHSR, including its constitutive activity. GHSR functions as a dynamic sensor of peripheral metabolic signals. Appetite regulation is also shaped by brain circuits involved in food reward and motivation**.** The activity of this system appears to be depend less on the absolute levels of ghrelin or LEAP2, and more in the relative balance between these hormones, reflected in the ghrelin/LEAP2 ratio [27, 159–161].

In conditions of nutritional deficit (e.g., fasting or starvation), circulating ghrelin levels rise while LEAP2 concentrations fall, resulting in an increased ghrelin/LEAP2 ratio that enhances GHSR activation [8, 27]. This shift promotes hunger, stimulates GH secretion, and supports adaptive responses to energy deficit, including increased food-seeking behaviour and energy conservation. Consistent with this physiological response, the restrictive eating disorder anorexia nervosa is characterized by chronically elevated ghrelin and desacyl-ghrelin levels and reduced LEAP2 concentrations, reflecting prolonged energy deprivation (Table 1) [161–163]. This hormonal profile results in a markedly increased ghrelin/LEAP2 ratio that would be expected to strongly activate GHSR signalling. Paradoxically, however, despite markedly elevated ghrelin concentrations, appetite and food intake remain suppressed. This observation raises the possibility that patients with anorexia nervosa develop a state of “ghrelin resistance”, potentially due to impaired responsiveness of central appetite-regulating circuits to ghrelin signalling, methylation of the GHSR promoter, and/or alterations in LEAP2 levels [164, 165]. An alternative explanation is that ghrelin signalling may simply not be sufficient or strictly necessary to drive these effects under certain pathological conditions, highlighting the need for further mechanistic investigation.

**Table 1:** Ghrelin/LEAP2 ratio in the context of nutritional states, obesity, and eating disorders

Condition	Ghrelin/LEAP2 ratio	GHSR Signalling	Behavioural/metabolic outcomes	Refs
Fasting	Ghrelin ↑/LEAP2 ↓ ↑ Ratio	Increased	Hunger, food seeking, GH secretion, adaptive response to energy deficit	[8, 27]
Feeding	Ghrelin ↓/LEAP2 ↑ ↓ Ratio	Decreased	Satiety and suppression of food intake	[8, 27, 71]
Anorexia nervosa	Ghrelin ↑/LEAP2 ↓ ↑ Ratio	Expected ↑ but centrally impaired	Persistent anorexia, weight and fat loss despite hyperghrelinemia (“ghrelin resistance”), reduced impulsivity (i.e. lower scores of impulse regulation) in refeeding	[161, 162, 262]
Cachexia	Ghrelin ↑/LEAP2 ↓ ↑ Ratio	Functionally reduced	Persistent anorexia and progressive weight loss	[263, 264]
Obesity	Ghrelin ↓/LEAP2 ↑ or = ↓ Ratio	Blunted	Reduced ghrelin responsiveness and impaired adaptive response to caloric restriction	[17, 31, 176]
Binge-eating disorder	Ghrelin ↓/LEAP2 = ↓ Ratio	Enhanced reward signalling	Food craving and loss of control during binge episodes	[159, 160, 163, 169]
Bulimia nervosa	Ghrelin ↑ (pre-binge)/LEAP2 ↓ ↑ Ratio	Increased	Facilitates binge episodes followed by post-binge ghrelin suppression	[170]
Prader–Willi syndrome	Ghrelin ↑↑ Unknown LEAP2 and Ratio	Increased, but without changes in GHSR expression	Hyperphagia, early-onset obesity	[166, 265]
PMOS	Ghrelin ↓/LEAP2 ↓ Unknown Ratio	Likely reduced GHSR activation	Reflects metabolic dysfunction and insulin resistance rather than changes in appetite	[19, 175]

GHSR, growth hormone secretagogue receptor; LEAP2, liver-expressing antimicrobial protein 2; PMOS, polyendocrine metabolic ovarian syndrome [formerly known as polycystic ovary syndrome (PCOS)]

Some studies further indicate that the ghrelin/LEAP2 ratio may exhibit dysregulated responses during prolonged caloric restriction and subsequent refeeding, which could contribute to unstable remission and difficulties in maintaining long-term weight recovery. In addition, emerging evidence suggests that ghrelin may influence neurocognitive processes involved in decision-making and reward evaluation, raising the possibility that altered ghrelin signalling contributes not only to metabolic dysregulation but also to the persistence of restrictive eating behaviours [162]. Similar alterations in ghrelin signalling have also been reported in cachectic states, in which circulating ghrelin concentrations are frequently elevated despite persistent anorexia, weight loss, and depletion of fat mass [166]. Reduced postprandial ghrelin levels and a lower ghrelin/LEAP2 ratio have also been described in amyotrophic lateral sclerosis, which may contribute to appetite suppression through reduced ghrelin action and increased ghrelin resistance [167]. Together, these patterns have been interpreted as compensatory responses to negative energy balance and may reflect impaired orexigenic signalling under severe catabolic conditions.

After feeding, ghrelin is rapidly suppressed and LEAP2 concentrations markedly increase, lowering the ghrelin/LEAP2 ratio and attenuating GHSR signalling, thereby promoting satiety and limiting further food intake [8, 27, 71]. In obesity, this reciprocal pattern also appears to be altered. Circulating ghrelin levels are typically lower than expected for the degree of adiposity [17], whereas LEAP2 concentrations remain relatively unchanged or may even be elevated [27, 31], a profile that results in a reduced ghrelin/LEAP2 ratio (Table 1). This shift may attenuate GHSR signalling and has been proposed to contribute to reduced ghrelin responsiveness, potentially impairing adaptive homeostatic responses during caloric restriction or weight loss attempts. Although the reduced ghrelin/LEAP2 ratio observed in obesity could theoretically favour increased ghrelin sensitivity by lowering chronic GHSR stimulation, current evidence overall supports the presence of obesity-associated ghrelin resistance [70]. This impaired responsiveness may arise through multiple mechanisms, including: (i) reduced circulating ghrelin levels; (ii) impaired transport across the blood–brain barrier; (iii) decreased hypothalamic expression of GHSR; (iv) suppression of AgRP/NPY neuronal activity due to hypothalamic inflammation, lipotoxicity, ER stress, and dysregulation of AMPK-mTOR signalling pathways; and (v) relatively increased LEAP2 levels, which further attenuate GHSR signalling [17, 27, 70, 168].

Analogously, binge-eating disorders have been associated with reduced circulating ghrelin levels. However, these levels show no correlation with the frequency of binge-eating episodes, suggesting that low ghrelin may represent a secondary adaptation aimed at counteracting the positive energy balance characteristic of this condition [160, 169]. Conversely, increased ghrelin levels have been found in individuals with bulimia nervosa, which may facilitate binge-eating behaviour, followed by a decrease after the binge episode [170]. This pattern may favour a ghrelin-dominant signalling state and enhance activation of neural circuits involved in food reward and motivation, potentially contributing to heightened food craving and loss of control during eating episodes. In a murine model of binge eating, plasma ghrelin and LEAP2 levels remain unchanged [159]. However, central administration of LEAP2 reduces binge-like intake of a HFD, an effect not observed with central ghrelin administration or with pharmacological blockade of GHSR using the antagonists [D-Lys3]-GHRP-6 and JMV2959.

Hyperghrelinemia is also a characteristic feature of Prader–Willi syndrome, in which elevated circulating ghrelin levels are detected from early childhood, even before the onset of hyperphagia, suggesting its potential participation in the characteristic hyperphagic phenotype and subsequent obesity [166]. Increased ghrelin levels are further reported from patients with hypothyroidism due to Hashimoto's disease [171], but not in people with the obesity-associated genetic disorder Bardet-Biedl syndrome [172] or Cushing's disease [173]. Women with polyendocrine metabolic ovarian syndrome (PMOS) [formerly known as polycystic ovary syndrome (PCOS)] [174], a condition frequently associated with adiposity and metabolic disturbances predisposing to diabetes, exhibit reduced ghrelin and LEAP2 levels in relation to the degree of insulin resistance [19, 175, 176]. However, the ghrelin/LEAP2 ratio in these conditions remain largely unexplored and may help explain the accompanying disturbances in appetite regulation.

Together, these observations highlight that the ghrelin/LEAP2 ratio may function as a metabolic and behavioural rheostat, regulating not only energy balance but also appetitive control and reward-related feeding behaviour. Its disruption across both ends of the nutritional spectrum (starvation and overnutrition) underscores its potential relevance to the pathophysiology of eating disorders. Future studies should determine whether restoring a physiological ghrelin/LEAP2 ratio could help normalize eating behaviour and energy regulation in these conditions, thereby bridging mechanistic endocrinology and clinical psychiatry.

## Glucose Homeostasis Control

Ghrelin is key for the maintenance of glycaemia during fasting. In this regard, both *Ghrl-* and *Ghsr-*knockout mice develop profound hypoglycaemia during starvation [78, 177]. Ghrelin regulates blood glucose through multiple targets involved in glucose handling [12], potentially to maintain glycaemia between meals. Overall, ghrelin tends to raise glycaemia by suppressing insulin secretion, reducing peripheral glucose uptake and insulin sensitivity, and increasing circulating glucocorticoids, glucagon release, and hepatic gluconeogenesis (Fig. 3). Beyond these peripheral actions, ghrelin also modulates glucose metabolism via hypothalamic–autonomic pathways that regulate hepatic glucose production and pancreatic hormone secretion [78]. By contrast, LEAP2 exerts glucose-lowering effects. High glucose levels stimulate hepatic LEAP2 production and secretion [157], and LEAP2 infusion lowers postprandial plasma glucose and GH concentrations [178] while enhancing insulin secretion [59].

### Regulation of Insulin and Glucagon Secretion in Pancreatic Islets

The pancreas expresses ghrelin, GOAT and GHSR, serving as a site for both local production and action of the ghrelin system [179–181]. Ghrelin is produced by pancreatic ε-cells (< 1% of the human islet cells) [180, 181], whereas GHSR is expressed in β- and δ-cells [182, 183]. In human islets, ghrelin and desacyl-ghrelin exert cytoprotective effects by promoting β-cell proliferation and inhibiting apoptosis [182] as well as by attenuating endoplasmic reticulum stress and inflammatory c-Jun N-terminal kinase (JNK) signalling induced by glucotoxicity [184]. Despite these protective actions, ghrelin contributes to increased blood glucose levels by inhibiting β-cell insulin secretion and stimulating α-cell glucagon secretion, effects that may also involve enhanced somatostatin release from δ-cells [75, 185–189]. This ability of ghrelin to regulate insulin and glucagon secretion through direct receptor-mediated actions in pancreatic islet cells may also represent part of the physiological glucoregulatory response to caloric restriction [188, 190].

Both ghrelin and LEAP2 modulate insulin and somatostatin secretion in an opposing and sex-dependent manner [191]. In rat pancreas, LEAP2 counteracts the insulinostatic effect of ghrelin [192]. Consistently, in a murine model of T2D, two weeks of LEAP2 administration ameliorated HFD-induced glucose intolerance and improved pancreatic islet morphology, alongside enhanced glucose-stimulated insulin secretion [193]. Moreover, a secreted endogenous LEAP2 fragment (LEAP2_38–47_) shows strong insulinotropic properties in vitro, enhancing insulin secretion in human pancreatic islets to a degree comparable to glucagon-like peptide 1 (GLP-1) [59]. Accordingly, another study found that LEAP2 was related to improved pancreatic insulin secretion in people living with overweight and obesity [64]. In humans, plasma LEAP2 concentrations decrease during glucagon infusions, whereas insulin appears to be required for postprandial LEAP2 upregulation following mixed-meal intake in mice [194]. Collectively, these findings highlight a complex interdependency between ghrelin, LEAP2, insulin, and glucagon signalling, which is further shaped by metabolic state and warrants additional investigation.

### Hepatic Glucose Metabolism and Peripheral Insulin Sensitivity

In rodents, ghrelin administration exacerbates hepatic gluconeogenesis and impairs insulin-mediated suppression of hepatic glucose output through modulation of AMPK-dependent pathways [195, 196]. In humans, plasma glucose levels increase after 2 h of ghrelin infusion, and this effect is markedly greater in people living with obesity than in those with healthy weight [197]. However, whether increased hepatic glucose production fully explains the rise in plasma glucose observed during ghrelin infusion in humans remains controversial [130, 131, 197]. In this context, ghrelin also impairs insulin sensitivity in the liver [195, 198] and skeletal muscle [130, 131]. In line with this, deletion of ghrelin in leptin-deficient obese *ob/ob* mice improves the phenotype of diabetes by increasing peripheral insulin sensitivity [75]. More favourable effects on glucose metabolism have been described for desacyl-ghrelin by enhancing hepatic and skeletal muscle insulin sensitivity [128, 199–201]. In patients with GH deficiency, acute ghrelin administration diminishes insulin sensitivity, while co-administration of ghrelin and desacyl-ghrelin markedly improves insulin sensitivity [202]. These findings overall suggest that ghrelin can impair glucose handling, while desacyl-ghrelin may partially counterbalance these insulin-desensitizing effects.

High glucose levels enhance LEAP2 production and secretion from the liver in mice and humans [27, 63, 203, 204]. It has been proposed that the hyperinsulinemia as opposed to high glucose levels, rather than glucose per se*,* drive the increased circulating LEAP2 concentrations [194]. In this regard, insulin receptor (INSR)- and glucagon receptor (GCGR)-expressing hepatocytes represent the main source of hepatic LEAP2 expression [194]. Current knowledge of the direct action of LEAP2 in hepatocytes remains scarce and controversial. *Leap2* knockdown has been reported to ameliorate hepatic steatosis and improve insulin sensitivity through restoration of IRS/AKT signalling in the liver of mice with diet-induced obesity [63]. Conversely, chronic central administration of LEAP2 attenuates hepatic inflammation and ER stress in mice with diet-induced obesity, although these protective effects appear to be blunted with ageing [112]. Thus, further studies are required to clarify whether LEAP2 can reverse ghrelin-induced impairments in hepatic insulin signalling and hepatic glucose production.

### Integration with Central Glucose-Sensing Circuits

In starvation (e.g. anorexia nervosa), cachexia and other negative energy balance states, ghrelin rises and central GHSR becomes essential for maintaining glycaemic control during severe energy deficit [205, 206]. In particular, GHSR-expressing AgRP neurons are not only key mediators of orexigenic effects of ghrelin, but are also sufficient to drive its glycaemic actions [78]. In line with this, *Ghsr*-knockout mice develop profound hypoglycaemia under caloric restriction, whereas selective re-expression of GHSR in AgRP [78] or hindbrain [207] neurons fully restores the normal glycaemic response to fasting. The glucoregulatory rescue in AgRP neurons is associated with increased glucagon secretion and induction of hepatic gluconeogenesis [78].

Another hypothalamic population implicated in glucose sensing are GHSR- and corticotropin-releasing factor (CRF)-expressing neurons in the paraventricular hypothalamus (hereafter named PVH^CRF^ neurons) [208]. During fasting, the hypothalamic–pituitary–adrenal axis is activated with PVH^CRF^ neurons secreting CRF into the median eminence. This, in turn, stimulates the secretion of adrenocorticotropic hormone (ACTH) from pituitary corticotrophs [209]. ACTH subsequently acts on the adrenal cortex to promote glucocorticoid secretion, which increase blood glucose levels through several mechanisms. Ghrelin stimulates hypothalamic–pituitary–adrenal axis through the activation of PVH^CRF^ neurons [210], with ghrelin treatment robustly increasing GH and glucocorticoid levels in rodents and in healthy people [211, 212]. During fasting, plasma ghrelin levels are increased [209, 213], whereas LEAP2 are dramatically decreased [23, 27, 208]. Interestingly, the fall of LEAP2 during fasting activates PVH^CRF^ neurons in a ghrelin-independent manner [208]. This suggests that enhanced GHSR signalling, driven not only by increased ghrelin but also by reduced LEAP2, may represent a physiologically relevant neuroendocrine mechanism supporting glucose homeostasis during prolonged fasting. However, emerging evidence indicates that LEAP2 may exert biological effects beyond GHSR antagonism. In GHSR-knockout mice under caloric restriction, chronic LEAP2 administration still promoted weight loss and hypoglycaemia [214], suggesting the involvement of GHSR-independent mechanisms. Together, these data highlight the need for further investigations to delineate the full spectrum of LEAP2-mediated receptor interactions, downstream pathways, and potential off-target effects.

### Pathophysiological Contexts and Clinical Correlates

The ghrelin-GHSR system contributes to the development of T2D by reducing insulin secretion, promoting insulin resistance, and enhancing hepatic glucose production [215]. Ghrelin exerts glucose-dependent insulin-suppressive effects in isolated islets from donors with T2D, but these islets contain approximately 75% fewer ghrelin-producing pancreatic ε-cells compared with non-T2D donors [189]. Consistently, in humans, ghrelin administration leads to insulin resistance independently of GH, cortisol, or FFA levels [131, 198]. Despite these hyperglycaemic actions, ghrelin exerts protective effects in several diabetic complications due to its anti-inflammatory, antioxidant, and anti-apoptotic properties. Preclinical evidence supports potential therapeutic application in diabetic chronic renal disease [216], cognitive impairment [217], wound healing [218], cardiomyopathy [219], retinopathy [220] and liver complications [20, 31]. This functional duality underscores the complexity and context dependence of therapeutic strategies targeting the ghrelin-GHSR axis in T2D.

LEAP2 acts as an endogenous antagonist of GHSR, counterbalancing the metabolic effects of ghrelin and demonstrating potential to improve glucose tolerance and β-cell function [215, 221]. Higher circulating LEAP2 levels are associated with improved insulin secretory function in individuals with overweight or obesity [64]. In line with this observation, serum LEAP2 is elevated in patients with insulinoma and correlates with hyperinsulinemia [222]. Elevated LEAP2 levels are also found in T2D [31] and MASLD [63], with fasting LEAP2 concentrations correlating positively with BMI, fasting plasma glucose, HbA1c, insulinemia, and indices of insulin resistance across diverse populations, including individuals with prediabetes, obesity, and T2D [27, 31, 66, 194, 204, 223], suggesting a close association between LEAP2 and metabolic dysregulation. Current management strategies for obesity and T2D include life-style interventions, pharmacotherapy, endoscopic and bariatric surgery procedures [224]. Notably, standard medical management, including a sustained 600-kcal/day energy deficit over 2 years, as well as duodenal-jejunal bypass liner (EndoBarrier®) therapy (removed after 1 year), have been associated with reductions in circulating LEAP2 compared with baseline levels [66]. In contrast, glucose-lowering interventions such as metformin, dapagliflozin, or structured exercise do not appear to modify LEAP2 concentrations in individuals with prediabetes [223]. Evidence regarding the impact of bariatric surgery on LEAP2 levels remains limited and inconsistent, with studies reporting decreased levels [27] or no significant changes [31, 225]. Collectively, these findings suggest that LEAP2 regulation may be more closely linked to energy balance and weight loss-related interventions than to glycaemic control per se. Further well-designed clinical trials will clarify the role of LEAP2 in T2D pathophysiology and to determine its therapeutic potential.

## Therapeutic Outlook and Translational Challenges

**Fig. 4:**
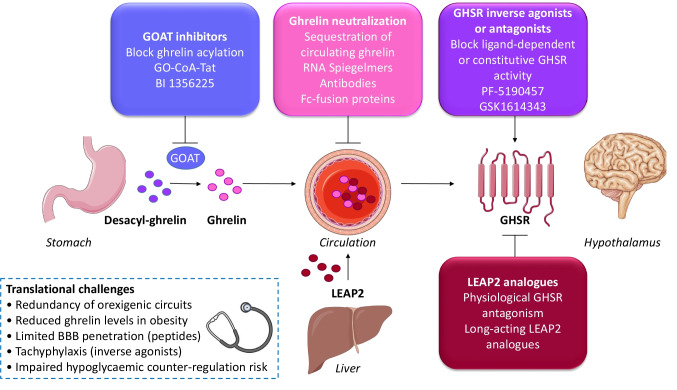
Pharmacological strategies targeting the ghrelin-LEAP2 system. Approaches include inhibition of GOAT-mediated ghrelin acylation, neutralization of circulating ghrelin, pharmacological blockade of GHSR ligand-dependent and constitutive activity, and long-acting LEAP2 analogues. Translational limitations include reduced ghrelin levels in obesity, limited CNS penetration of peptide-based therapies, redundancy of orexigenic circuits, tachyphylaxis, and potential impairment of fasting counter-regulatory responses

Pharmacological approaches targeting the ghrelin system to modulate appetite and metabolism include ghrelin antagonists, GOAT inhibitors, GHSR inverse agonists and LEAP2 analogues (Fig. 4) [226, 227]. These compounds act by blocking or attenuating ghrelin activity, or by promoting its natural antagonist, LEAP2. GOAT inhibitors prevent the final acylation step of ghrelin required for ghrelin activation, while GHSR inverse agonists block both ghrelin-stimulated and constitutive (basal) receptor activity. Despite promising preclinical findings, these approaches face several challenges, including the reduced ghrelin levels found in obesity, limited peptide stability, difficulties in central drug delivery, potential off-target effects, and the need to establish long-term efficacy and safety [226, 227]. Nevertheless, the ghrelin-LEAP2 axis may be more useful for preventing weight regain, controlling binge eating, or improving glucose counter-regulation, rather than producing large primary weight loss.

### Neutralization of Ghrelin

Spiegelmers (from the German *Spiegel*, meaning “mirror”) are synthetic nucleic acids engineered to bind specifically to target molecules [228]. Spiegelmers contain *D*-ribose instead of the naturally occurring *L*-ribose, rendering them resistant to nuclease degradation and highly stable in biological fluids for 60 h or longer. The anti-ghrelin Spiegelmer NOX-B11 constitutes a polyethylene glycol-modified *L*-RNA oligonucleotide that specifically binds to ghrelin, thereby preventing its interaction with GHSR [229]. Acute administration of NOX-B11 inhibits ghrelin-induced food intake and c-Fos induction in the rat ARC [228]. Chronic infusion of NOX-B11 in mice with diet-induced obesity blocks ghrelin-induced food intake and reduces body and fat mass [230], while also preventing weight regain during fasting-refeeding cycles [231]. Although Spiegelmer NOX-B11 has been suggested as a potential therapeutic option for conditions associated with hyperghrelinemia, such as the Prader-Willi syndrome [228], current evidence remains limited to preclinical studies, and clinical trials are required to validate its efficacy and safety in humans. Clinical neutralization of ghrelin with vaccines, antibodies, or Spiegelmers often fails to translate into significant weight loss due to redundancy within hypothalamic orexigenic circuits (including AgRP/NPY pathways) and compensatory adaptations that preserve feeding behaviour [232, 233].

### GOAT Inhibitors

Inhibition of GOAT, the membrane-bound enzyme responsible for ghrelin acylation, can be achieved by targeting the substrate-binding site, the co-substrate (acyl donor) site, or by using bisubstrate mimetics [234, 235]. Intraperitoneal administration of GO-CoA-Tat, a peptide-based bisubstrate analogue, ameliorates glucose tolerance and reduces weight gain in mice [236]. In rats, pharmacological GOAT blockade with GO-CoA-Tat reduces food intake during the dark phase by enhancing satiety, while satiation remains unaffected [237]. BI 1356225 is a novel orally available small-molecule GOAT inhibitor that has progressed to Phase I randomized, placebo-controlled clinical trials, demonstrating good tolerability in healthy males and in individuals with overweight or obesity [238]. Although BI 1356225 reduces circulating ghrelin levels and decreases the ghrelin/desacyl-ghrelin ratio by > 80% over 28 days of treatment, it does not produce measurable effects on body weight, hunger or satiety ratings, eating behaviour, or energy intake. These data suggest that a reduction of ghrelin using GOAT inhibitors is not sufficient to induce clinically relevant body weight loss.

### GHSR antagonists/Inverse Agonists

Pharmacological inhibition of GHSR using antagonists or inverse agonists has shown promise for ameliorating obesity-related metabolic disorders. Central administration of the selective GHSR antagonist JMV2959 suppresses both ghrelin-induced and fasting-induced food intake by regulating ghrelin-responsive ARC neuron activity, and also reduces motivation to obtain palatable foods such as sweets [94, 239]. Beyond antagonism of ligand-dependent signalling, GHSR inverse agonists target the constitutive (basal) receptor activity. In this regard, treatment with the inverse agonists GHSR-IA1 and IA2 improves glucose homeostasis and metabolic outcomes in Zucker diabetic *fa/fa* rats and mice with diet-induced obesity [240], likely through enhanced glucose-dependent insulin secretion [241]. However, due to the dual nature of GHSR signalling—constitutive basal activity and ghrelin-mediated ligand activation—effective modulation may require a combined strategy using both an antagonist and an inverse agonist [221]. PF-5190457 was the first GHSR inverse agonist developed, capable of blocking both endogenous ghrelin action and constitutive receptor activity. Intracerebroventricular administration of PF-5190457 attenuates ghrelin-induced food intake in male mice only, but reduces HFD-induced binge-eating behaviour in both sexes [242]. Importantly, PF-5190457 has advanced into clinical development and is well tolerated after oral administration in humans [243]. In clinical studies, PF-5190457 has also been shown to reduce alcohol-related food craving in people with alcohol use disorder [244], highlighting potential applications beyond classical metabolic indications. Of note, chronic dosing of PF-5190457 can induce tachyphylaxis. By contrast, intermittent short-term central blockade may preserve efficacy and reduce tachyphylaxis [243]. Despite these promising findings, translation of GHSR antagonists and inverse agonists from preclinical models to clinical trials remains challenging. This is largely due to the complexity of GHSR signalling and the need for high selectivity to avoid off-target effects and unwanted adverse outcomes [245]. An additional translational concern is that chronic inhibition of GHSR signalling may impair counter-regulatory responses during prolonged fasting, potentially increasing vulnerability to hypoglycaemia in susceptible individuals.

### GHSR Agonists

In veterinary medicine, ghrelin receptor agonists are already in clinical use as potent appetite stimulants to promote weight gain, although they have not yet been approved for metabolic indications in humans. Capromorelin (also referred to as AT‐002, RQ‐5, and CP‐424,391), was the first GHSR agonist to receive FDA approval for the stimulation of appetite in dogs [246]. The therapeutic potential of GHSR agonists has been investigated for the management of anorexia, weight loss, sarcopenia, and cancer cachexia [153–155, 247]. In this regard, GHSR agonist anamorelin was approved in 2020 in Japan for the treatment of cachexia in patients with non-small cell lung cancer, gastric cancer, pancreatic cancer, and colorectal cancer [248]. Although anamorelin improves appetite and body weight, it has not been approved by the US Food and Drug Administration, largely because of its limited efficacy in improving muscle strength and physical function. These limitations highlight the need for next-generation GHSR agonists with greater efficacy in preserving skeletal muscle mass and function in models of aging and cachexia [249].

### GHSR-independent actions of ghrelin and LEAP2

Several studies support the existence of an alternative, yet unidentified, receptor mediating the actions of desacyl-ghrelin [250]. Although the corticotropin-releasing factor type 2 receptor (CRF2R) has been proposed as a potential mediator of some desacyl-ghrelin effects [251], this hypothesis remains controversial [252]. Nevertheless, desacyl-ghrelin has been shown to regulate skeletal muscle metabolism, bone formation, and cardioprotection through mechanisms that appear to be independent of canonical GHSR signalling [253]. In addition, emerging evidence suggests that LEAP2 may also exert GHSR-independent actions. Continuous LEAP2 administration to calorie-restricted *Ghsr*-knockout mice induces weight loss, hypoglycaemia, reduced body temperature, and increased hepatic IL-6 and IL-1β mRNA expression, indicating that LEAP2 can modulate physiological processes beyond the ghrelin-GHSR system [214]. Collectively, these findings suggest that both desacyl-ghrelin and LEAP2 may act through additional signalling pathways outside canonical GHSR activation, thereby broadening their potential therapeutic implications and identifying important areas for future investigation.

### LEAP2 Analogues

Infusion of native LEAP2 to supraphysiological concentrations reduces food intake and lowers postprandial plasma glucose to a magnitude comparable to native GLP-1 in healthy men, without inducing nausea or other gastrointestinal adverse effects [178]. However, native LEAP2 exhibits a short half-life (10–15 min in rodents and approximately 9 min in humans), which limits its therapeutic utility [23, 254, 255]. To overcome this, several long-acting (LA) LEAP2 analogues have been developed, employing strategies such as lipidation or other chemical modifications to enhance stability. For instance, LA-LEAP2 analogues demonstrate a half-life exceeding 5 h in mice, enabling more practical dosing regimens, including daily or potentially weekly administration. Subcutaneous administration of palmitoylated LEAP2(1–14) variant for 8 weeks reduces ghrelin-induced c-Fos immunoreactivity in the ARC and area postrema and attenuates hepatic de novo lipogenesis, but does not prevent weight gain in diet-induced obesity [256]. Notably, a degree of resistance to the anti-obesity effects of palmitoylated LEAP2(1–14) has been observed in mice fed a high-fat diet, an effect that is reversed upon switching to a standard chow diet [257]. By contrast, another lipidated LA-LEAP2 compound reduces both body weight and food intake while preserving energy expenditure in mice with diet-induced obesity [254]. Collectively, these findings indicate that LEAP2 analogues can achieve modest weight reduction (~ 10%) and improve markers of liver inflammation in preclinical models of obesity [255]. A recent study demonstrated that continuous intravenous LEAP2 infusion over 5 h reduces both glycaemia and food intake in men living with obesity, supporting the therapeutic potential of LEAP2 for obesity and related metabolic disorders [258]. Nevertheless, the efficacy of LEAP2 monotherapy does not match that of GLP-1 receptor (GLP-1R) agonists, suggesting that combination therapies or further optimization may be required to realize their full therapeutic potential [221].

### Combination with GLP-1R Agonists

The glucagon-like peptide-1 receptor (GLP-1R) and GHSR trigger opposing effects on food intake. Activation of GLP-1R promotes potent appetite-suppressing effects, while activation of GHSR robustly stimulates feeding behaviour. Consequently, GHSR blockade could potentially modulate the anorexigenic and weight-reducing actions of GLP-1R agonists. Acute pharmacological inhibition of GHSR, either through intraperitoneal administration of the GHSR antagonist JMV2959 or intracerebroventricular treatment with LEAP2, did not enhance the reduction in food intake observed 36 h after liraglutide treatment in mice [259]. However, combined administration of semaglutide with a long-acting LEAP2 analogue for two weeks produced a more pronounced decrease in whole-body adiposity and attenuated weight regain 14 days after treatment cessation [254].

## Conclusions and Future Perspectives

The ghrelin-LEAP2 system has emerged as a central regulatory system coordinating energy balance, glucose homeostasis, and behavioural responses to nutritional status [91, 194, 206, 221, 260, 261]. By integrating peripheral metabolic signals with hypothalamic neuroendocrine circuits, ghrelin and its endogenous antagonist LEAP2 dynamically regulate feeding behaviour, glucose counter-regulation, and metabolic adaptation to fasting and nutrient availability. Disruption of this balance contributes to the pathophysiology of obesity, T2D, and maladaptive feeding behaviours, positioning the ghrelin-LEAP2 system as a biologically compelling yet complex therapeutic target [113, 161].

Despite substantial advances in understanding ghrelin signalling over the past 25 years [1], no GHSR-targeted therapy has yet translated into clinical practice. The discovery of LEAP2 as an endogenous GHSR antagonist has reignited interest in this pathway as a therapeutic target for obesity and/or T2D [59, 139, 254, 259], but important mechanistic gaps remain. In particular, the physiological role of LEAP2 beyond GHSR antagonism, the tissue-specific actions of ghrelin and desacyl-ghrelin, and the functional relevance of the ghrelin/LEAP2 ratio across different metabolic states require further clarification. Moreover, the redundancy and plasticity of central appetite-regulating circuits, together with the constitutive activity of GHSR and the development of tachyphylaxis, complicate pharmacological modulation of this system.

Future research should focus on elucidating ghrelin-LEAP2 interactions to uncover previously unrecognized regulatory mechanisms and enable the development of novel therapeutic strategies. Rather than serving as a primary driver of weight loss, modulation of ghrelin-LEAP2 axis may prove particularly valuable for improving metabolic resilience during energy deficit, preventing weight regain after weight reduction, and modulating reward-driven feeding. Continued integration of mechanistic, translational, and clinical research will be essential to determine whether targeting the ghrelin-LEAP2 system can complement current metabolic therapies and contribute to more effective strategies for the treatment of obesity and related metabolic derangements.

## Key References

- Cui H, López M, Rahmouni K. The cellular and molecular bases of leptin and ghrelin resistance in obesity. Nat Rev Endocrinol. 2017;13:338–51.
  o Of outstanding importance
  o This seminal review summarizes the central and peripheral mechanisms underlying ghrelin resistance in obesity, including hypothalamic inflammation, impaired ghrelin transport, and altered GHSR signalling. It provides an essential conceptual framework for understanding dysregulated ghrelin responsiveness in metabolic disease.
- Tezenas du Montcel C, Duriez P, Cao J, Lebrun N, Ramoz N, Viltart O, et al. The role of dysregulated ghrelin/LEAP-2 balance in anorexia nervosa. iScience. 2023;26:107,996.
  o Of outstanding importance
  o This study highlights the potential role of an altered ghrelin/LEAP2 ratio in the pathophysiology of anorexia nervosa. The findings support the concept of “ghrelin resistance” and suggest that dysregulation of this hormonal axis may contribute to persistent restrictive eating behaviours and impaired weight recovery.
- Stark R, Feehan J, Mousa A, Andrews ZB, de Courten B. Liver-expressed antimicrobial peptide 2 is associated with improved pancreatic insulin secretion in adults with overweight and obesity. Diabetes Obes Metab. 2023;25:1213–20.
  o Of importance
  o This study links circulating LEAP2 concentrations with improved pancreatic insulin secretion in adults living with overweight or obesity. The findings suggest that LEAP2 may participate in glucose homeostasis beyond its antagonistic effects on ghrelin signalling.
- Johansen VBI, Gradel AKJ, Holm SK, Cuenco J, Merrild C, Petersen N, et al. Regulation of LEAP2 by insulin and glucagon in mice and humans. Cell Rep Med. 2025;6:101,996.
  o Of importance
  o This study identifies insulin and glucagon as key regulators of LEAP2 expression and secretion in mice and humans, providing important mechanistic insight into the integration of the ghrelin–LEAP2 system with glucose homeostasis. The findings establish LEAP2 as a metabolically regulated hormone closely linked to nutrient sensing and endocrine control of energy balance.
- Englund A, Lange AH, Hagemann CA, Kizilkaya HS, Rosenkilde MM, Hartmann B, et al. LEAP2 reduces ad libitum food intake and attenuates postprandial glucose excursions in men with obesity. Diabetes. 2026:10.2337/db25-1132.
  o Of outstanding importance
  o This study provides the first direct clinical evidence that LEAP2 infusion reduces food intake and improves postprandial glucose control in men living with obesity. These findings strongly support the translational and therapeutic potential of targeting the ghrelin–LEAP2 system in metabolic disease.
- Holm SK, Johansen VBI, Ranea-Robles P, Svendsen C, Merrild C, Rohlfs R, et al. Sustained weight loss with combined LEAP2 and semaglutide treatment in mice. Diabetes. 2025;74:2089–100.
  o Of outstanding importance
  o This preclinical study demonstrates that combined LEAP2 and semaglutide treatment produces sustained weight loss and attenuates weight regain in obesity. The work supports the potential of combining ghrelin–LEAP2 modulation with GLP-1 receptor agonism as a novel anti-obesity strategy.

## Data Availability

No datasets were generated or analysed during the current study.

## Abbreviations

ACC: Acetyl-CoA carboxylase
AgRP: Agouti-related peptide
AMPK: AMP-activated protein kinase
ARC: Arcuate nucleus
BAT: Brown adipose tissue
C/EBPα: CCAAT/enhancer-binding protein α
ChREBP: Carbohydrate responsive element-binding protein
CPT1: Carnitine palmitoyl-transferase 1
CRF: Corticotropin-releasing factor
DGAT1: Diacylglycerol O-acyltransferase 1
DMH: Dorsomedial hypothalamic nucleus
DRN: Dorsal raphe nucleus
ER: Endoplasmic reticulum
FASN: Fatty acid synthase
FFA: Free fatty acid
GOAT: Ghrelin-*O*-acyl transferase
HFD: High-fat diet
GABA: γ-Aminobutiric acid
GH: Growth hormone
GHSR: Growth hormone secretagogue receptor
GLP-1: Glucagon-like peptide 1
LEAP2: Liver-expressed antimicrobial peptide 2
LHA: Lateral hypothalamic area
LPL: Lipoprotein lipase
MASLD: Metabolic dysfunction-associated steatotic liver disease
MOGAT2: Monoacylglycerol O-acyltransferase 2
NPY: Neuropeptide Y
PMOS: Polyendocrine metabolic ovarian syndrome
POMC: Proopiomelanocortin
PPARγ: Peroxisome proliferator-activated receptor γ
PVH: Paraventricular hypothalamic nucleus
ROS: Reactive oxygen species
SCN: Suprachiasmatic nucleus
SIRT1: Sirtuin-1
SREBF1: Sterol regulatory element-binding protein 1
T2D: Type 2 diabetes
UCP1: Uncoupling protein 1
VMH: Ventromedial nucleus oh hypothalamus
VTA: Ventral tegmental area
WAT: White adipose tissue
